# Mentalization-based interventions in schools for enhancing socio-emotional competencies and positive behaviour: a systematic review

**DOI:** 10.1007/s00787-024-02578-5

**Published:** 2024-09-12

**Authors:** Gali Chelouche-Dwek, Peter Fonagy

**Affiliations:** Psychoanalysis Unit, Research Department of Clinical, Educational and Health Psychology, University College London, Gower Street, London, WC1E 6BT UK

**Keywords:** Mentalization, School-based intervention, Socio-emotional development, Children, Adolescents, Systematic review

## Abstract

Mentalization-based interventions (MBIs) have been increasingly applied in school settings to support the social-emotional development and mental health of children and adolescents. This systematic review aimed to synthesize the evidence on the effectiveness of MBIs implemented in educational contexts for students aged 6–18 years. A comprehensive search was conducted in PsychInfo, MEDLINE, EMBASE, Web of Science, and ERIC databases from inception to October 2023. The search strategy combined terms related to mentalization, school-based interventions, and the target age group. The review protocol was registered with PROSPERO (CRD42022302757). Inclusion criteria included peer-reviewed publications in English, studies published between 1980 and 2023, interventions based on mentalization principles, and a primary focus on children aged 6 to 18 years. Exclusion criteria involved non-mentalization based interventions and research outside the 6–18 age range. The risk of bias was assessed using the Quality Assessment Tools from the National Institutes of Health (NIH). Data were synthesized narratively due to the heterogeneity of study designs and outcomes. Of the 5,250 articles screened, 21 studies met the inclusion criteria, comprising over 7,500 participants. The reviewed interventions targeted various aspects of mentalizing, such as emotion-understanding, empathy, perspective-taking, and Theory of Mind. Significant improvements were found in social-cognitive abilities, emotion regulation, and mental health outcomes, including reductions in disruptive behaviours. Interventions that combined mentalizing training for both students and teachers showed promising results. However, the long-term sustainability of these benefits remains unclear. Limitations of the reviewed studies include the lack of control groups, small sample sizes, and variations in outcome measures. The findings highlight the potential of MBIs as a promising approach to fostering socio-emotional competence, positive behaviour, and well-being in school-aged children. Future research should aim to establish the active components and optimal delivery of these interventions through well-designed randomized controlled trials with larger, more diverse samples and extended follow-up periods. The integration of MBIs within educational systems holds promise for promoting resilience and positive mental health outcomes in young people. Embedding MBIs within school curriculums and evaluating cost-effectiveness are important next steps to guide widespread implementation.

## Introduction

### Mentalization-based treatments

Mentalizing refers to the process of understanding one’s own and others’ actions as manifestations of mental states such as thoughts, emotions, and intentions [1]. This ability typically develops within the context of an attachment relationship, closely linked to the caregiver’s capacity to interpret and respond to the child’s internal experiences [2]. Humans instinctively form assumptions about others’ mental states, and these assumptions significantly influence our own thoughts and behaviours. Developing robust mentalizing abilities during childhood is crucial for cultivating social-emotional skills such as empathy, emotional regulation, perspective-taking, and effective navigation of social interactions [3, 4].

Nonetheless, some children experience challenges in mentalizing, often stemming from attachment disruptions, traumatic experiences, or developmental and learning difficulties [5]. These challenges can adversely affect their interpersonal relationships, self-regulatory capabilities, and academic achievement, and increase the risk of developing psychological disorders [6]. Furthermore, a compromised capacity to mentalize can hinder effective coping mechanisms, adaptation, and resilience in stressful situations [7]. Impaired mentalizing abilities may result in impulsive behaviour, aggression, and other maladaptive responses. Situational loss of mentalizing capacity can occur under stress. Additionally, this capacity might be significantly reduced or absent in individuals with various psychopathologies [1, 2].

Mentalization-Based Treatment (MBT), developed to enhance resilience in the ability to mentalize, was first introduced in the 1990s as a therapeutic strategy for individuals diagnosed with Borderline Personality Disorder (BPD) [1]. It is based on the understanding that a tendency towards frequent interruptions in mentalizing and a delayed restoration of mentalizing in social interactions is a central problem in BPD [8]. MBT incorporates elements from both psychodynamic and cognitive-behavioural approaches to create a comprehensive therapeutic framework, aiming to improve the patient’s mentalizing capacity, especially within the framework of attachment relationships. From the cognitive-behavioral perspective, MBT incorporates techniques aimed at enhancing the client’s ability to observe and understand their own mental states and those of others. This includes the use of psychoeducation and cognitive restructuring to address distorted thinking patterns, structured exercises that help clients identify and challenge thoughts and beliefs, thereby promoting more adaptive thinking patterns and behaviours. For example, clients might engage in role-playing scenarios to practice and reinforce mentalizing skills in social contexts, which is a hallmark of cognitive-behavioural interventions [2, 5]. Also, MBT practitioners apply techniques designed to foster mentalizing, guided by several principal strategies such as managing anxiety to ensure it remains within a range conducive to mentalizing, as excessive or insufficient anxiety can obstruct the mentalizing process, fostering a relational process by transitioning from focusing on individual mentalizing towards a collective understanding or “we-mode” in the therapeutic relationship and prioritising the process of how mental states are understood over the detailed examination of content that is not well mentalized [5]. By adhering to these MBT principles, therapists foster an environment conducive to mentalizing. This enhancement of mentalization is theorised to lead to improved emotion regulation, impulse control, self-awareness, and social interactions [5].

Over the past twenty years, MBT has been adapted for various settings, including family therapy, individual therapy, and group therapy. It has also been integrated into treatments for a diverse range of clinical issues, such as substance abuse, antisocial personality disorder [9], eating disorders [10] and depression [11]. Also, therapeutic strategies incorporating mentalizing principles include for example the Reflective Parenting Program, which focuses on enhancing parental reflective functioning [12], Mentalization-Based Treatment for Adolescents (MBT-A) that incorporates mentalizing principles to help young people understand and regulate their emotions, improve their relationships, and reduce self-harm and suicidal behaviours [2, 13], and Mentalization-Based Treatment for Adolescents with Conduct Disorder (MBT-CD) to improve mentalizing capacities, reduce conduct problems, and enhance overall psychological functioning [14]. These approaches focus on enhancing the adolescent’s ability to mentalize within the context of attachment relationships, particularly with caregivers and peers. Importantly, while the structure of MBT can be tailored to different contexts, its foundational principles and the general framework of the treatment have remained consistent.

### Mentalizing-based interventions in schools

The successful application of MBT in adult populations led to the adaptation of MBT for children, adolescents, and families soon after the model’s introduction [10]. Recent therapeutic strategies have incorporated mentalizing principles to develop interventions aimed at enhancing mentalizing abilities in young people.

While MBT is a structured treatment, usually lasting for 12–18 months, and is defined by a set of techniques as detailed in the previous section, mentalization-based interventions (MBIs) are specific interventions that are focused on aspects related to the ability to mentalize - to reflect on the mental states of self and others. More specifically, these interventions typically include activities designed to increase awareness of mental states and their link to behaviour, such as psychoeducation, exercises in identifying emotions, discussions about the motivations behind actions, metaphorical storytelling, and reflective conversations about real-life social situations [2, 11]. These approaches have shown clinical effectiveness in managing conditions like borderline personality disorder and self-harm [9, 12], as well as conduct disorders [13].

The encouraging results from MBT in clinical settings have led to its application in non-clinical environments, particularly in schools, over the past two decades. Researchers from various psychological fields have focused on school-based interventions, repeatedly finding positive effects on mental health and academic performance. These interventions, grounded in a range of theoretical frameworks and addressing diverse objectives, have included group Cognitive Behavioural Therapy (CBT) sessions [14], parenting training interventions for conduct problems [15], initiatives targeting childhood and adolescent obesity [16], and strategies for understanding and addressing bullying dynamics [17]. Schools have proven to be a suitable settings for these interventions due to their capacity for wide-reaching impact. Given the extensive amount of time children spend in school, these settings offer a convenient and cost-effective platform for delivering preventative interventions at scale and with high potential for broad applicability [18].

Within the framework of MBIs in schools, it is essential to underscore that the primary emphasis of these interventions is on augmenting the ability to contemplate the mental states of oneself and others. This focus on reflection distinguishes mentalization-based approaches from other social-cognitive interventions, marking it as a key element of the intervention strategy. In the past two decades, various initiatives have adapted mentalizing techniques for educational settings to enhance children’s socioemotional growth proactively, demonstrating the effectiveness of MBIs in reducing violence, bullying, and antisocial behaviour among students [11, 19–21].

The importance of mentalizing skills in the effectiveness of educators for different age groups has been discussed in both theoretical and empirical frameworks [21– 23]. The ability of teachers to comprehend their own and their students’ mental states, emotions, motivations, and viewpoints is pivotal in creating an effective educational environment, catering to the diverse needs of all students [24]. Mentalization theory offers a valuable framework for teacher training and school-based interventions, with a focus on nurturing reflective abilities and fostering positive relationships. Developing these mentalizing skills among school staff and students is instrumental in transforming the school atmosphere and facilitating student development [21, 22].

MBIs likely facilitate change through various mechanisms. At a fundamental level, discussing mental states raises awareness that behaviours are often reflections of internal subjective experiences, not merely rigid traits or unexplained idiosyncrasies [25]. Teachers embody this understanding through their curiosity about students’ motivations, coupled with providing validation and support. Gradually, students adopt mentalistic approaches to comprehend their own and others’ actions. Enhanced mentalizing skills aid in managing emotional states and resolving conflicts in a constructive manner, as opposed to resorting to aggression [2]. As mentalizing within the classroom improves, these environments become more secure and empathetic, reinforcing the students’ belief that their teachers understand them, which is beneficial for their learning. Furthermore, when teachers adopt a mentalizing approach, they can build stronger connections with even the most challenging students, mitigating their own adverse reactions such as burnout or harsh disciplinary actions that can emerge when mentalizing is impaired [26].

### The present study

In the sphere of child development, recent research has increasingly focused on infants, preschool-aged children, and their parents, illuminating the crucial role of parental reflective functioning in fostering secure attachments. This newfound insight has spurred the creation of early-years MBIs. Prominent among these are programs like Minding the Baby [27], Reflective Parenting [28] and Mothering from Inside Out [29], which concentrate on nurturing parental mentalizing abilities.

Despite advancements in MBIs for parents and young children, as well as for children and adolescents in various contexts such as family therapy [30] and individual therapy [31], existing related systematic reviews such as MBT and its evidence-base status [32], MBT for children aged 6–12 and their carers [33], and for children and families [34], have indicated a lack of similarly developed interventions in school settings, interventions for adolescents and highlighted the need for further research in non-clinical settings. While the value of reflective functioning is increasingly acknowledged in educational contexts, there seems to be a notable void in the availability of school-based mentalization interventions specifically designed for this age range. This gap presents a significant opportunity for further research and the development of interventions, which could enhance our comprehension and implementation of mentalization approaches in schools, thereby addressing the diverse needs of children across different age brackets.

The preliminary research suggests that MBIs implemented in school settings offer considerable promise in enhancing social-cognitive skills that are essential for positive psychosocial development during crucial formative years. The current research literature lays the groundwork for considering mentalization as a viable method for alleviating various forms of psychological distress and behavioural issues among young people. Additionally, MBIs are increasingly being utilized in educational environments, not only to address maladaptive behaviours in children but also to cultivate a positive and productive learning atmosphere [11]. However, to date, there appears to be no comprehensive systematic review that has evaluated the different types of MBIs conducted in school settings and assessed their overall effectiveness.

The proposed systematic literature review aims to appraise the effectiveness of a variety of MBIs in schools, taking into account their potential limitations. The focus will be primarily on empirical studies of school-based MBIs that offer quantitative data on their efficacy or effectiveness. This review intends to amalgamate evidence from various school-based mentalization programs to ascertain their influence on outcomes such as socioemotional skills, conduct problems, academic performance, and classroom environment. Furthermore, the review will culminate with a discussion on how the findings from this systematic review could inform policy-making and practical decisions regarding the adoption and refinement of MBIs in educational settings. This synthesis of knowledge is crucial for supporting the evidence-based implementation of emerging mentalization frameworks in schools, aiming to foster the well-being and overall potential of students.

### Objective of the systematic review

The objective of this systematic literature review is to gain insight and evaluate of the impact of various MBIs in school environments. The review is guided by the following specific objectives:

1. **Identification and Analysis of Research Gaps**: To pinpoint and scrutinize the existing gaps in the current body of research on MBIs within school settings, particularly for children aged 6–18. This involves assessing the scope and depth of current studies and identifying areas lacking sufficient research.
2. **Synthesis of Empirical Evidence**: To compile and present a comprehensive resource for both academics and practitioners by collating and analysing empirical evidence from studies on school-based MBIs. This synthesis aims to provide clear insights into the effectiveness and efficacy of these interventions in educational settings.
3. **Evaluation of Outcomes**: To evaluate the effectiveness of school-based mentalization programs on various outcomes such as socioemotional skills development, reduction in conduct problems, enhancement of academic performance, and improvement in classroom climate.

This systematic review is significant in its aim to foster the evidence-based application of emerging mentalization frameworks in school settings. By doing so, it contributes to enhancing the well-being and unlocking the potential of students across a broad spectrum of age groups, thereby addressing a critical aspect of educational and psychological development.

## Method

### Search strategy

The methodology of this systematic review adheres to the Preferred Reporting Items for Systematic Reviews and Meta-analyses (PRISMA) guidelines [32]. The review protocol, inclusive of analytical methods and selection criteria, was duly registered with the International Prospective Register of Systematic Reviews, known as PROSPERO (Registration number: CRD42022302757). An initial comprehensive literature search was conducted in January 2022, followed by a subsequent search in October 2023. This two-phased approach aimed to encompass both existing and recent studies, focusing on the exploration of various MBI types implemented in school settings and assessing their effectiveness.

The search targeted several key databases, namely PsychInfo, MEDLINE, EMBASE, Web of Science, and ERIC (Educational Resources Index). To ensure a focused yet comprehensive search, specific terms were used, as detailed in Table 1 of the review document.

To manage and streamline the collation of relevant literature, duplicate articles were systematically removed using the EndNote Web tool. Furthermore, to capture a wider range of pertinent research, the reference lists of the identified studies, along with relevant reviews and meta-analyses, were scrutinized for additional relevant studies. This comprehensive search strategy is designed to ensure the inclusion of a wide array of studies, thereby enhancing the robustness and depth of the systematic review.

**Table 1:** Electronic search terms

Search Term Category	Terms Applied
***Population***	(school* or education* or pupil* or teacher*)
***Intervention***	(therap* or intervention* or treatment* or project*)
***Outcomes***	(mentali* or “reflective function*” or mind-minded* or mindedness or alexithymia or “emotional recognition” or “theory of mind” or “social cognition”)

### Inclusion and exclusion criteria

The inclusion and exclusion criteria for this systematic review were carefully defined to ensure a focused and relevant analysis of the literature on MBIs in school settings. The criteria are as follows:

#### Inclusion criteria:

1. **Peer-Reviewed Publications in English**: Only peer-reviewed studies in English were included to ensure the credibility and quality of the research.
2. **Publication Period**: Studies published between 1980 and 2023 were considered, allowing for a comprehensive overview of the development and application of MBIs over time.
3. **Types of Interventions**: The interventions investigated in these studies had to be based on mentalization or closely associated theoretical frameworks. This included interventions promoting empathy, reflective functioning, social cognition, and Theory of Mind.

Interventions focusing on the capacity to improve understanding behaviour of others or the self in terms of mental states were included in the review.

4. **Target Age Group**: The primary focus of the interventions should be on children aged 6 to 18 years, meaning at least 90% of participants had to be in this age range.
5. **School-Based Programs**: were expected to be based in schools or, if not conducted in a school were directly relevant to and explicitly aligned with school-based practices [33].
6. Outcome measures: Studies that report quantitative measures of outcomes relevant to CYP development, mental health, wellbeing or function.

#### Exclusion Criteria:

1. **Non-Mentalization Based Interventions**: Studies focusing on interventions not primarily informed by mentalization theory or its related frameworks were excluded.
2. **Scope of Study**: Articles and book chapters predominantly dealing with neurological or physiological aspects rather than the psychological or educational application of MBIs were excluded.
3. **Age Group Limitations**: Research exclusively focusing on populations outside the 6–18 age range, such as children under 5 or adults over 19, was not considered.
4. Type of publication: We excluded reviews, commentaries, opinion pieces, conference abstracts, study protocols, experimental studies and dissertations.

By adhering to these criteria, the review aims to collate and analyse a body of research that is both relevant and rigorous, offering insights into the effectiveness and application of MBIs in educational settings for the specified age group. The inclusion of studies based on a broad but related set of theoretical frameworks ensures a comprehensive understanding of the field, while the exclusion criteria help maintain the review’s focus on the most pertinent and high-quality research.

### Data extraction

Independent researchers carried out data extraction. All records identified were uploaded to Endnote 20 (Team, 2013) and deduplicated following the process set out by Bramer and colleagues [34]. All titles and abstracts were screened using Rayyan, with records clearly not meeting inclusion criteria excluded [35]. Full texts of remaining records were then reviewed, with reasons for exclusion noted for all studies. 75% and 100% of records were double screened by two independent reviewers at the title and abstract stage and full text stage, respectively. All conflicts were taken forward to the full text stage. This systematic approach was bolstered by an updated literature search in October 2023, complementing the initial search conducted in January 2022. All articles that conformed to the inclusion criteria were earmarked for an in-depth full-text review. Disagreements at the full text stage were resolved through discussion with a third reviewer. Additionally, an exhaustive examination of the reference lists in selected studies, alongside pertinent meta-analyses and review articles, was undertaken to uncover any additional relevant literature. This step ensured a comprehensive coverage of the topic. Decisions regarding the eligibility of studies were reached through consensus, following in-depth discussions where necessary.

Data from each study meeting inclusion criteria was extracted by two reviewers into an excel based form. Information extracted was then compared to reach consensus. Data extracted included: the authors, year of publication, country of origin, age range, sample size, study design (pre-post/ RCT/ quasi-expiramental), targeted demographic group, the format of the intervention, the principal findings and outcomes, and were collated by the first author. Effect sizes were also extracted where available, and in studies where they were not reported, efforts were made to estimate them from the available data.

Owing to the diversity and extensive range of the identified studies, conducting a meta-analysis was deemed not feasible. The variations in study populations, the outcomes measured, and the diversity of measurement methods, coupled with a quantity of studies insufficient for a robust meta-analysis, necessitated an alternative approach for data synthesis. Consequently, the synthesis of data is presented in a narrative format, organized with regard to the various dimensions of mentalizing addressed and the main findings. This narrative approach allows for a detailed and nuanced examination of the studies while accommodating their inherent heterogeneity.

### Quality assessment

The assessment of study quality was conducted using the Quality Assessment Tools provided by the National Institutes of Health (NIH), which are publicly available on the NIH website. Two different tools were applied separately for controlled and uncontrolled studies. Specifically, the tools used included the NIH Quality Assessment Tool for Observational Cohort and Cross-Sectional Studies and the NIH Quality Assessment Tool for Controlled Intervention Studies. The first author, in collaboration with an experienced research colleague, independently carried out these evaluations. This approach allowed for a thorough and unbiased assessment, as both evaluators brought different perspectives and expertise to the analysis.

Each study was evaluated for several quality parameters as outlined in the NIH’s tools. These parameters included, but were not limited to, the study design, methodological rigour, sample size, appropriateness of the statistical analyses, clarity in reporting results, and the relevance of the findings to the research questions. The independent nature of the assessments by the two evaluators helped in providing a balanced view of each study’s quality.

In instances where there were differences of opinion regarding the quality ratings, these were resolved through detailed joint discussions. Such discussions were instrumental in reaching a consensus, thereby ensuring that the final quality assessment was reflective of a comprehensive and collaborative evaluation. This consensus-based approach not only fortified the reliability of the quality assessment but also added depth to the evaluation process by combining diverse viewpoints and analytical skills.

### Data synthesis

Included studies showed significant variation in the information reported (for example, only reporting percentages, without providing the overall number of students this related to), the conditions being studied, and the timeframe of data collection. It was therefore unlikely that an average estimate across studies would be of clinical use [36]. Furthermore, not all studies reported the necessary raw data or sample sizes required to compute effect sizes or confirmed the independence of sub-groups. A narrative synthesis was therefore conducted.

## Results

The systematic search across multiple electronic databases initially yielded a total of 8,458 studies. When the pre-established inclusion and exclusion criteria were applied, this number was reduced to 6,953 studies. Further refinement through the removal of duplicate entries led to a pool of 5,250 studies. A detailed screening of these studies, focusing on their titles and abstracts, resulted in the exclusion of a significant number – 5,141 studies were deemed not relevant to the scope of this review. Consequently, 109 studies were identified as potentially relevant based on their abstracts. However, upon a full-text review, 92 of these studies were excluded due to various reasons such as not meeting the specific criteria or lacking sufficient depth in MBIs.

During the process of reviewing the full texts, an exhaustive examination of the citations and reference lists from the eligible studies was also conducted. This led to the identification and inclusion of an additional 4 articles that met the review criteria. The entire selection process, following the rigorous standards set by the Preferred Reporting Items for Systematic Reviews and Meta-analyses (PRISMA) guidelines, was conducted with a high degree of thoroughness and collaborative discussion amongst the authors. Any uncertainties or ambiguities encountered during the study selection were resolved through these discussions, ensuring a robust and consensus-based approach.

**Fig. 1:**
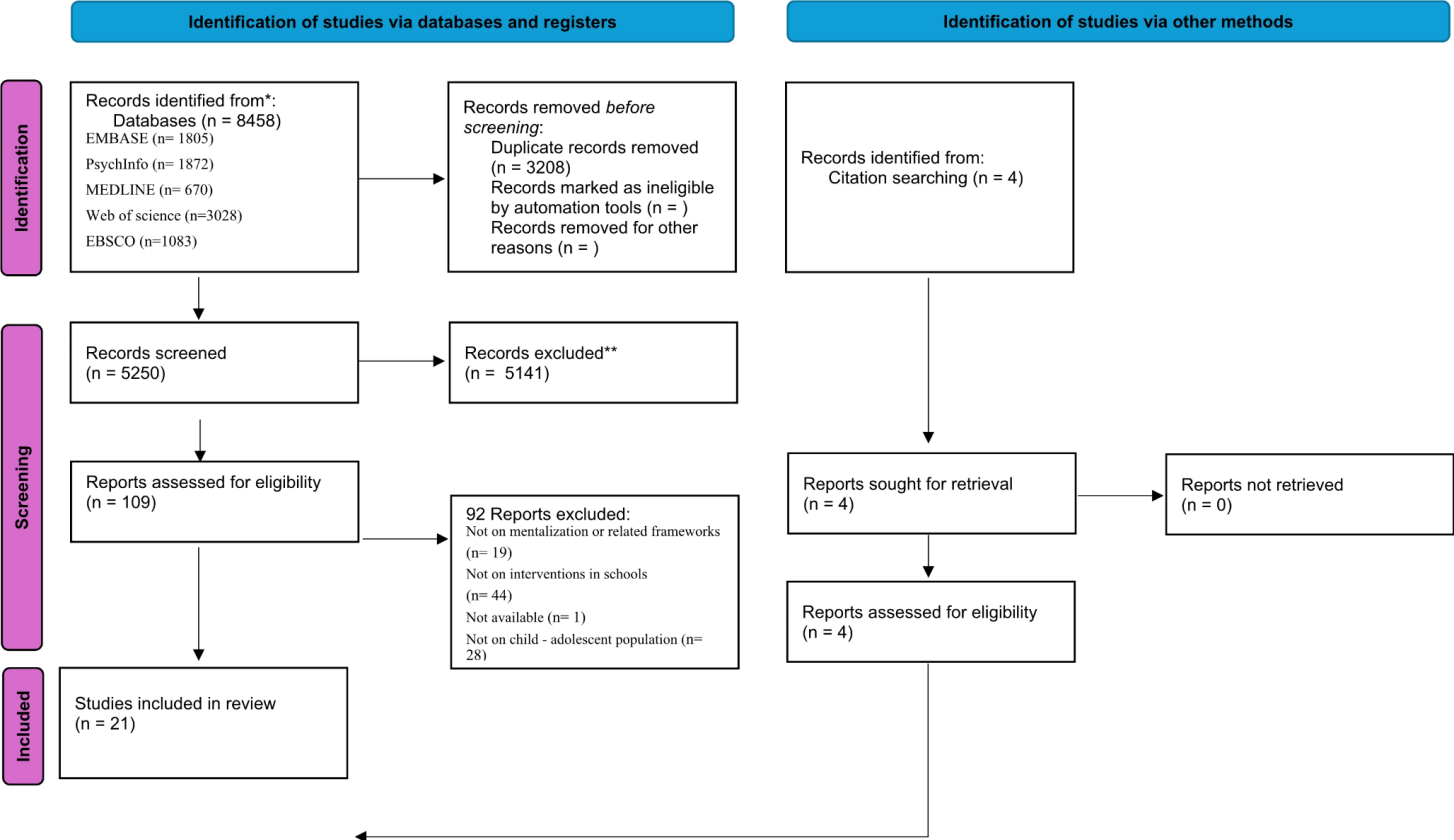
PRISMA Flow Diagram Illustrating the Study Selection Process. *From*: Page MJ, McKenzie JE, Bossuyt PM, Boutron I, Hoffmann TC, Mulrow CD, et al. The PRISMA 2020 statement: an updated guideline for reporting systematic reviews. BMJ 2021;372:n71. doi: 10.1136/bmj.n71.

Ultimately, after this comprehensive evaluation, 21 articles were deemed suitable for inclusion in the systematic review. The flow of information through the different phases of this systematic review is graphically represented in Fig. 1. This figure provides a visual summary of the screening process, illustrating the step-by-step reduction and refinement of studies, culminating in the final selection of articles for the review.

**Table:** 

Author (year)	Country	Age group	Sample size	Study design	Intervention name	Target beneficiary group(s)	Aspect of mentalization	Main significant findings	Outcomes	Effect size	QA
Bak et al. (2015)	Denmark	adolescents	130	Pre-post pilot study	The Resilience Program	Students, the staff, and the broader community.	Mentalization	3 years after the intervention, 80–100% of staff were still actively using components of the Resilience Program in their work. The program was rated as very valuable by staff. The frequency of violent incidents decreased after the intervention as did staff sick days.	Child violent incidents (*p* < .05) Carer sick days (*p* < .05)	Child violent incidents: Pre: rate ratio 4.36, 95% CI [2.41–8.56] Post: rate ratio 2.28, 95% CI [1.37–3.92]	Fair
Bianco et al. (2016)	Italy	8–9 years old	72	Quasi-experimental	ToM conversation-based training	Students	ToM	ToM training group improved significantly more than control group on ToM skills after training and at 2 month follow up.	Child ToM skills Post: (*p* < .001) Follow up: (*p* = .028)	The ToM group outperformed the control one at post-test Post: d = 0.79 Follow up: d = 0.42	Good
Caputi et al. (2021)	Italy	9–10 years old	210	RCT	ToM conversation-based training	Students	ToM	ToM training group obtained higher ToM and lower loneliness scores at post-test compared to control. However, loneliness effect was not sustained at the 2 month follow up.	Child ToM skills (*p* < .001) Child loneliness scores (*p* = .003)	ToM skills: d = 0.55	Good
Doron E. (2016)	Israel	9–13 years old	150	Quasi-experimental	Creativity and divergent thinking training	Students	Mindfulness, ToM, perspective taking	Children who participated in the program scored significantly higher on the divergent thinking tests after the intervention took place and showed higher creative achievements compared to children in the control groups.	Child creative achievements: fluency (*p* = .012) and uniqueness (*p* < .001)	Fluency: pη2 = 0.05 Uniqueness: pη2 = 0.11	Good
Eppler-Wolff et al. (2020)	USA	7-year-old girl (Marie) and a teacher in her late 50’s (Ms. Henry).	-	Qualitative two case studies	School-Based Mental Health Collaboration (SBMHC)	Students and teachers	Mentalization	Discussed role of nested mentalization in stakeholder support. Did not have control group at follow-up. The cases illustrate SBMHC model can improve reflective functioning and mentalization capacity for both students and teachers through the nested mentalization framework.			NA
Finne et al. (2017)	Norway	11–14 years old	332	Pre-post	Social Perception Training (SPT)	Students	Social cognition, perception, interpretation	Reduction in cognitive distortions and problem behaviours, improvement in social skills and perceived learning environment.	Child cognitive distortions (*p* < .0001) Child behaviour problems (*p* < .0001) Child social skills (*p* < .0001) Child perceived learning environment (*p* = .010)	η2 = 0.11 η2 = 0.09 η2 = 0.08 η2 = 0.02	Fair
Fonagy et al. (2009)	USA	8–11 years old	1345	RCT	Peaceful Schools Program	Students	Mentalization	The CAPSLE intervention significantly reduced peer-reported aggression, victimization, aggressive bystanding, improved empathic mentalizing compared to control. It also reduced disruptive/off-task classroom behaviours.	Child peer-reported aggression (*p* < .05) Child peer-reported victimization (*p* < .01) Child aggressive bystanding (*p* < .05) Child empathic mentalizing (*p* < .01) Child disruptive/off-task classroom behaviours (*p* < .05)		Good
Greenberg et al. (1998)	USA	5–12 years old	57	Quasi-experimental	PATHS (Promoting Alternative Thinking Strategies) curriculum	Deaf children	Emotional awareness, understanding others’ perspectives	Intervention group showed significant improvements in students’ social problem-solving skills, emotional recognition skills, and teacher- and parent-rated social competence.	Child social cognition (*p* < .05) Child emotional recognition (*p* < .001) Child positive social competence (*p* < .05)		Good
Iuso et al. (2022)	Italy	12–14 years old	191	Pre-post	Psychoeducational program	Students prone to bullying behaviours	Empathy, alexithymia	Psychoeducational program significantly reduced alexithymia levels, increased empathy, and emotion regulation capacities among adolescents.	Child alexithymia (*p* = .0001) Child Empathy (*p* = .0092) Child Emotional regulation (*p* < .0001)		Fair
Karray et al. (2020)	Tunisia	8–12 years old	105	Quasi-experimental	“Game of Three Figures” intervention	Students	Empathy, alexithymia	“Game of Three Figures” intervention increased empathy compared to control group.	Child Empathy (*p* < .05)		
Lecce et al. (2014)	Italy	9–10 years old	91	RCT	ToM conversation-based training	Students	ToM	Compared with the control group, children in the experimental group performed significantly better on the ToM task at post-test and follow-up and made greater gains in ToM from pre-test to post-test and from pre-test to follow-up.	Child ToM task performance (*p* = .001)	pη2 = 0.11	Good
Lombardi et al. (2021)	Italy	8–10 years old	110	Quasi-experimental	ToM conversation-based training	Students	Perspective-taking, ToM, reflective thinking	The training group showed significant improvements in altruistic behaviour and investment decisions compared to the control group.	Child altruistic behaviour (*p* = .022) Child investment decisions (*p* = .041)	η2 = 0.071 η2 = 0.038	Good
Lombardi et al. (2022)	Italy	7–8 years old	56	RCT	Thoughts in Mind - Child (TiM-C)	Students	Emotion regulation, metacognition, ToM	Significant improvements over the training period only in the TiM-C Project group for Metacognition, Emotion Regulation Strategies and a ToM task.	Child metacognition (*p* < .001) Child emotion regulation (*p* = .011) Child ToM (*p* < .001)	pη2 = 0.23 pη2 = 0.13 pη2 = 0.29	Good
Oker et al. (2020)	France	9–11 years old	22	Quasi-experimental	Interaction with a virtual tutor agent named “Alice”	Students	Empathy	Students were more accurate and took longer to respond correctly when interacting with the bimodal (facial expressions) agent. They also rated this agent as more empathetic.	Child more accurate response times (*p* < .04) Child rated agent as more empathetic (*p* < .01)	pη2 = 0.20 d = 1.76	Good
Ornaghi et al. (2014)	Italy	6–8 years old	110	Quasi-experimental	Conversational intervention on emotions	Students	Emotion understanding, ToM, empathy	The trained group outperformed the control group on post-test measures of emotion understanding, theory of mind, and empathy (affective domain of empathy showed no difference). The positive effect of the intervention on emotion understanding remained stable 6 months later.	Child emotion understanding (*p* = .0001) Child ToM (*p* = .0002) Child cognitive empathy ( *p* < .05 )	pη2 = 0.14 pη2 = 0.12 pη2 = 0.05	Good
Ratcliffe et al. (2014)	Australia	7–13 years old	217	Quasi-experimental	Emotion-Based Social Skills Training (EBSST)	Children with ASD	Emotional understanding, empathy, ToM	EBSST improved teacher-reported emotional competence in students, with a large effect size that was maintained at follow-up. No significant improvements on parent-reported outcomes or more general measures of social skills and mental health.	Carer emotional competence (*p* < .05)	η2 = 0.18	Good
Sagkal et al. (2012)	Turkey	6th grade elementary school students.	281	Quasi-experimental	Peace Education Program	Students	Empathy	The peace education program was effective in increasing students’ empathy levels compared to the control group. Both boys and girls in the intervention group showed increased empathy.	Child empathy (*p* < .05)	η2 = 0.17	Good
Twemlow et al. (2005)	USA	elementary/primary school students grade K-5.	3600	Quasi-experimental	Peaceful Schools Program	Students	Mentalization	Compared to control schools, the Peaceful Schools intervention showed significant reductions in peer reported victimization, aggression, aggressive bystanding, disruptive/off-task classroom behaviours over 2 years. Effects on victimization, aggression and aggressive bystanding maintained at 1 year follow-up.	Child peer-reported victimization (*P* < .01) and aggression (*P* < .05) and aggressive bystanding (*P* < .01) Child disruptive/off-task behaviours (*P* < .001)		Fair
Twemlow et al. (2001)	USA	5–10 years old	609	Quasi-experimental	Peaceful Schools Program	Students	Mentalization	The experimental school showed significant decreases in disciplinary referrals and suspensions. The experimental school showed increases in academic achievement test scores. The control school showed little change.	Child disciplinary referrals (*p* < .05) Child suspensions (*p* < .004) Child academic achievement test scores (*p* < .05)		Good
Twemlow et al. (2011)	Jamaica	12–15 years old	2 schools	Quasi-experimental	Peaceful Schools Program	Students	Mentalization, power dynamics, bullying	Improvements across 3 years in academic performance, decreased victimization, increased helpfulness, and the school becoming a place teachers wanted to work, and students wanted to succeed.	Child academic performance (*p* < .05) Child victimization (*p* < .05) Child helpfulness (*p* < .05)		Fair
Valle et al. (2016)	Italy	10 years old	46	Quasi-experimental	Thought in Mind (TiM) Project training	Students	Mentalization, ToM	Children of teachers who received TiM training improved more than controls in 3rd order false belief and mentalizing styles.	Child mentalizing (false belief understanding, Strange Stories, Reading the Mind in the Eyes, Mentalizing Task) (*p* < .05)	pη2 = 0.17–0.46	Fair

### Characteristics of the included studies

Table 1 provides a comprehensive summary of the studies reviewed, detailing specifics such as the authors, countries of origin, years of publication, and demographic data including age range and sample size. It also outlines the methodological attributes like study design, the facets of mentalizing addressed, the nature of the intervention, the target groups, and the principal findings.

#### Geographical distribution

The 21 studies included in the review represent a wide geographical spread, conducted across 9 countries. The bulk of these were carried out in Italy (8 studies), followed by the USA (5 studies), and single studies from Australia, Denmark, France, Israel, Jamaica, Norway, Turkey, and Tunisia. Such a broad international representation serves to enhance the generalizability and applicability of the findings across different cultural and educational contexts.

#### Study populations

The age groups targeted by these studies varied considerably, ranging from early elementary to early secondary school students. Both mainstream educational settings and special needs populations were included, indicating a broad interest in applying mentalizing interventions across diverse learning environments. A significant majority (18 studies) focused on typically developing students, including some in challenging environments such as violent or under-resourced schools [19–21, 37–41]. Additionally, one study specifically targeted children with Autism Spectrum Disorder (ASD) [42], and another was conducted with deaf children [43]. This indicates that while the majority of research on school-based mentalizing interventions has focused on universal prevention for general student bodies, there is a growing interest in targeted interventions for clinical subgroups, suggesting an area ripe for further investigation.

The collective sample size of over 7,500 children and adolescents across these 21 studies underscores the significant reach and potential impact of mentalizing interventions delivered through school settings. Such interventions demonstrate the capacity to engage large numbers of youth, particularly those who may not traditionally have access to additional counselling or psychological services. This extensive reach is critical for fostering widespread improvements in mentalizing abilities and related psychosocial outcomes among diverse student populations.

### Aspects of mentalizing targeted

The aspects of mentalizing targeted within the selected studies were varied, exemplifying the multifaceted nature of this concept. The studies endeavored to bolster several components of mentalizing, including empathy, Theory of Mind (ToM), emotional comprehension, perspective-taking, and mindfulness. A notable number of these studies primarily focused on enhancing ToM [37, 40]. This diversity in focus underscores the comprehensive approach of the interventions in addressing mentalizing.

Each intervention was uniquely designed to cater to the specific mentalizing components relevant to its target population. This tailored approach ensures that the interventions are appropriately aligned with the distinct needs and developmental stages of the students involved. For example, in younger children, where foundational aspects of ToM are still developing, interventions may concentrate more on fostering basic understanding of emotions and perspectives of others. In contrast, with older children or adolescents, the emphasis might shift towards more complex aspects of mentalizing, such as understanding nuanced emotional states and advanced perspective-taking.

The broad spectrum of mentalizing aspects addressed in these studies reflects an understanding of the complex interplay of cognitive and emotional processes in social interactions and personal development. By focusing on diverse components of mentalizing, these interventions aim not only to enhance the immediate social and emotional competencies of students but also to lay a groundwork for their long-term psychological well-being and interpersonal success. This approach is particularly relevant in educational settings, where fostering a range of mentalizing skills can significantly impact students’ academic and social experiences.

### Outcomes

Of the 21 studies identified by the review, four were randomized controlled trials (RCTs) [19, 40, 44, 45], whilst 13 had a quasi-experimental design. Although these quasi-experimental studies can provide valuable insights, the absence of an experimental design means that potential for confounding cannot be ruled out, which limits the inferences that may be drawn from the findings. Among the RCTs, one study [19] was notable for its relatively high-quality evidence, demonstrating significant reductions in peer-reported aggression and victimization, improved empathic mentalizing, and decreased disruptive/off-task classroom behaviours. This study, along with another RCT designs [44], which showed significant improvements in Theory of Mind (ToM) task performance, underscores the potential for mentalization-based interventions to provide robust evidence of positive impact.

Of the non-controlled studies, one paper reported results of a small qualitative evaluation [39], while most were simple pre-post intervention cohort studies, without a comparison group. These studies often demonstrated significant improvements in targeted outcomes, such as reductions in violent incidents [33] and alexithymia levels [41], although the lack of control groups means that natural fluctuations and external factors could not be discounted as explanation of observed benefit.

The assessment of study quality involved an examination of the study design, randomisation, sample size, and duration of follow-up. Although some studies incorporated control groups, the use of semi-random or non-random assignment to groups makes it challenging to establish causal links between interventions and outcome. A further recurrent issue was the underpowered designs with small sample sizes [21, 46], which affects the ability to generalise findings and highlights the necessity for larger-scale studies. Follow-up durations were frequently brief [44, 47], which leaves open the possibility of rapid reversal of observed benefits. Certain researchers have pointed out the provisional character of their findings [33] and highlighted the importance of replication, the need for recruitment of more diverse samples [19] and the ongoing refinement and evaluation of bespoke programmes. No study was compliant with Open Science guidelines with fully prespecified statistical modelling approaches and outcomes expectations.

Across the 21 evaluation studies, a wide range of outcomes were assessed. The results for each study are presented in detail in Table 1. A noteworthy finding from this review was the consistent significant improvement in children’s mentalizing capacities across several studies, although most of them did not set out measures of mentalizing as primary outcomes per se. Significant enhancements ToM Skills Improvement was presented in 5 studies post-intervention, demonstrating the impact of MBI on cognitive and affective ToM development [37, 40, 44, 45, 47]. In 3 studies, interventions led to a reduction in peer-reported aggression and victimization [11, 19, 20], and 2 studies reported improvement in emotional regulation capacities among children [41, 45].

The Peaceful Schools project [20, 25] included children’s self-reported experiences of and beliefs about aggression and victimization using various measures. One study explicitly attempted to measure the impact of the intervention on children’s mentalizing capacity [21], assessing this outcome using multiple measures (false belief understanding, Strange Stories, Reading the mind in the Eyes, Mentalizing Task). The results were mixed, showing positive intervention effects on only a few of these measures.

Most studies assessed outcomes for children in one or more domains. Common measures included child behaviour, wellbeing, and social skills. Almost all studies that assessed children’s general wellbeing found at least some positive changes over the intervention period.

It should be noted that the outcomes relating to child well-being were often carer-, clinician-, or teacher-reported. This reliance on indirect reporting can introduce bias and may not fully capture the child’s perspective. Two studies [25, 33] included children’s self-reported experiences of the beliefs about aggression and victimization using the Peer Experiences Questionnaire [48], which showed robust intervention effects.

Some studies aimed to support children’s wellbeing indirectly by targeting parents, carers, teachers, or staff. For these approaches, outcomes for the caregivers themselves, alongside carer-reported outcomes for the children, were often assessed. Several studies assessed caregivers’ mentalizing capacity using various measures. Only a few studies showed clear evidence of improved caregiver mentalizing from pre- to post-intervention [39, 41], with others finding no strong evidence for intervention effects in this domain. Additional outcomes for caregivers included measures of parenting stress and caregiver self-efficacy. Improvements in these areas were reported in a few studies, highlighting the potential secondary benefits of mentalization-based interventions.

In term of effect sizes, findings reveal that mentalizing-based interventions generally lead to medium to large effect sizes across various psychological and social outcomes. ToM conversation-based training consistently showed medium to large effect sizes, highlighting its efficacy in enhancing cognitive and social abilities. Notably, some studies reported very large effects for specific outcomes, such as perceived empathy (d = 1.76; 46). However, it’s important to note that not all studies reported standardized effect sizes, and the metrics used varied across studies, making direct comparisons challenging. Other mentalizing, school and social cognition programs, though less consistent in reporting effect sizes, demonstrated significant reductions in aggressive behaviours and improvements in mentalizing capacities.

Overall, the reported effect sizes suggest that MBIs are effective in fostering mentalization, empathy, social competence, emotional regulation, and academic performance among students. The reported effect sizes underline the substantial impact these programs can have, reinforcing their importance in educational and developmental contexts. Notably, structured trainings reveal relatively moderate to large effect sizes, demonstrating the robust potential of these interventions to significantly enhance various developmental outcomes in educational settings.

#### Theory of mind conversation-based training interventions

A number of studies have focused on implementing ToM training programmes, which are anchored in discussions about mental states, and was shown to significantly improve ToM skills among students. These programmes have been introduced in various educational settings, primarily targeting primary school children. For example, a ToM training programme consisting of four sessions was carried out, facilitated by primary school teachers for 72 children aged 8–9 years [37]. This programme combined individual work and group discussions centred around mental states, drawing upon short stories as a basis. The findings indicated that the group receiving ToM training showed significantly more improvement in ToM task performance compared to a control group, both immediately following the training and at a follow-up two months later. They reported that the ToM group outperformed the control one with a moderate effect size for ToM skills in post-intervention (d = 0.79, 95% CI [0.96, 3.16]) for the differences.

In a similar vein, a ToM training that involved 91 children aged 9–10 years [44], included conversations about mental states, using stories and activities focused on mental state verbs across four sessions, each lasting 50 min. The study included a control group that engaged in discussions about physical events. The children in the ToM group demonstrated notably greater progress in advanced ToM tasks, with a moderate effect size (η2 = 0.11, *p* = .001), both from the start to the end of the training and in the two-month follow-up.

Another study that was conducted in Italy involved 210 students aged 9–10 years in a similar ToM conversation-based training programme [40]. The outcomes measured were ToM abilities and feelings of loneliness. Post-training, the ToM group not only achieved higher scores in ToM but also reported lower levels of loneliness compared to the control group. However, these improvements were not observed in the follow-up after two months.

The scope of their conversational training programme was extended to include perspective-taking and reflective thinking, alongside ToM, for 110 primary school children in Italy [49]. The results of this training showed an improvement in altruistic behaviours (η2 = 0.071, *p* = .022), and investment decisions (η2 = 0.038, *p* = .041) among the participants compared to those in the control group.

More recently, a randomized controlled trial with 56 primary school children in Italy was undertaken, evaluating the Thoughts in Mind Project for Children (TiM-C) intervention [45]. The TiM-C programme, which spanned four weeks, involved stories and activities designed to teach children about mental states and strategies for regulating emotions. Assessments conducted before and after the training evaluated the children’s metacognition, emotion regulation strategies, and ToM abilities. Compared to the control group, students who participated in the TiM-C training exhibited significant improvements with large effect sizes in metacognitive skills (pη2 = 0.23, *p* < .001), emotion regulation (pη2 = 0.13, *p* = .01), and ToM (pη2 = 0.29, *p* < .001), as evidenced by their performance in a triangle animations task.

The cumulative findings from these randomized controlled trials consistently demonstrate that conversation-based training focused on mental states and perspectives can lead to significant improvements in social cognition, such as ToM, even in middle childhood. Moreover, ToM conversation-based training interventions demonstrated effect sizes ranging from medium to large, indicating substantial improvements in cognitive and social abilities among participants. The real-world application of these interventions in educational settings underscores their practicality and potential for wider application. Furthermore, the benefits of these interventions extend beyond ToM, contributing to positive social behaviours such as altruism and enhanced self-control capacities. This underscores the significant role of school-based ToM interventions in fostering socio-emotional competence among children.

#### Other social cognition training programs

In addition to ToM, several studies have assessed general social cognitive training programs based on mentalizing principles. These studies have explored diverse aspects of social cognition in various educational settings and have demonstrated significant positive outcomes. For example, The Social Perception Training (SPT) program was implemented in Norwegian primary and secondary schools [50]. This 10-week program aimed to enhance students’ social information processing abilities. Involving 18 classrooms and over 330 students, their pre-post study indicated that the SPT program led to a reduction in cognitive distortions and externalizing problems. Additionally, there were improvements in social skills and students’ perceptions of the school climate.

A randomized trial of a 10-week creativity program designed to foster perspective-taking and mentalizing in children aged 9–13 years was conducted in Israel [38]. The post-intervention results showed significant enhancements in divergent thinking compared to a control group, suggesting that the benefits of the training extended into the realm of creative cognition.

Expanding to a different developmental group, The Emotion-Based Social Skills Training (EBSST) program was evaluated, which was delivered over 16 weeks to 217 children with Autism Spectrum Disorder (ASD) enrolled in mainstream Australian schools [42]. The EBSST targeted emotional understanding, problem-solving, regulation, and coaching skills, with separate training sessions for students, parents, and teachers. While no changes were reported by parents, teachers observed significant and sustained improvements in emotional competence among the EBSST participants.

In Turkey, a Peace Education Program with 158 sixth-grade students, focusing on empathy, conflict resolution, and peaceful living skills was evaluated [51]. The post-intervention assessment showed that participants exhibited increased empathy compared to control groups.

Lastly, the impact of empathy expressed by virtual tutors on the learning of 22 elementary students was investigated in France [46]. A virtual agent that displayed empathic facial expressions led to an increase in students’ academic performance compared to an agent that only provided verbal feedback. This study highlights the potential benefits of integrating emotional signals into educational technologies.

These studies collectively provide preliminary evidence that school-based social cognitive interventions grounded in mentalizing theory, particularly those targeting perspective-taking, can enhance socio-emotional capacities like emotion recognition and regulation, social skills, cognitive flexibility, and classroom climate. This evidence spans a range of developmental periods and contexts. Overall, the direct enhancement of social cognitive capacities through structured interventions appears to be a promising approach, warranting further exploration and validation through more comprehensive controlled trials, including long-term follow-ups.

#### Targeting emotional understanding via conversational training

In the context of MBIs, four studies have focused on enhancing emotional understanding, empathy, and emotion regulation among elementary school children using conversational training methods. These interventions aimed to deepen children’s emotional comprehension and empathetic skills, crucial components of mentalizing.

In emotional conversational training over two months with 110 typically developing Italian primary school students [47], the children engaged in guided discussions about emotions after listening to emotionally charged stories. In contrast, the control group participated in activities such as drawing pictures related to the stories. The study found that compared to the control group, the training group showed significant improvements in emotion understanding, Theory of Mind (ToM), and cognitive empathy. Notably, the positive effects on emotional understanding were still evident six months later.

In a similar vein, in a program in Tunisia focused specifically on cultivating empathy through theatrical role-playing games [52], the intervention carried out over a school year, resulted in increased empathy among elementary students who participated in the theatre-based games, in comparison to those in the control group.

Recently, an eight-session psychoeducational program targeting 191 secondary school students aged 12–14 years was conducted [41]. This program was designed to enhance emotion regulation capacities and reduce bullying behaviours. Significant outcomes included reductions in alexithymia – a difficulty in recognizing and describing emotions – and improvements in empathy and emotion regulation skills, such as cognitive reappraisal. This finding aligns with existing literature suggesting that improved emotional awareness and regulation can play a crucial role in preventing bullying and enhancing socio-emotional competence in adolescents.

Adding a developmental dimension to these interventions, the Promoting Alternative Thinking Strategies (PATHS) curriculum, which focuses on emotion understanding and regulation among 57 deaf elementary school children at risk of psychosocial problems was conducted [43]. The PATHS intervention group exhibited significant improvements in social cognition, reduced impulsivity, and increased positive social behaviour and competence, compared to the control group. Follow-up assessments conducted one and two years later indicated that these gains were maintained or even augmented over time.

Synthesizing the findings across these studies, it is evident that conversational training and psychoeducational programs aimed at improving emotional understanding, empathy, and regulation can effectively enhance socio-emotional capacities. This is true not only for typically developing elementary schoolers but also for adolescents facing behavioural or developmental challenges. The benefits of these interventions span emotion comprehension, ToM, cognitive empathy, and social competence. Particularly noteworthy is the reduction of bullying risk factors, such as alexithymia, through psychoeducation. The successful implementation of these programs among deaf children further attests to their feasibility and applicability across diverse student populations. These interventions underscore the importance of directly targeting core aspects of mentalizing, including understanding emotions, as a means to support the social-emotional learning and peer interaction skills of young people.

#### Teacher training in mentalizing-based strategies

In the context of MBIs within educational settings, research has predominantly focused on student-centred approaches. However, one notable study stands out for its emphasis on training teachers in mentalizing strategies to enhance these capacities among their students.

The critical role of the Thoughts in Mind (TiM) teacher training program in enhancing mentalizing capacities among students was investigated through a randomized controlled trial [21]. This trial included 46 children aged 10 years and their teachers, with the objective of bolstering the teachers’ mentalizing skills. The TiM intervention comprised two sessions, each lasting three hours, where teachers were introduced to and trained in mentalizing concepts and techniques.

A particularly significant aspect of this study was its comprehensive assessment of mentalizing abilities in children. The evaluations, conducted before and after the intervention, included the Strange Stories task for assessing the application of Theory of Mind (ToM) in everyday social contexts [53], and the Reading the Mind in the Eyes Test-Child Version [54]. The findings were revealing: only the students whose teachers participated in the TiM Project training demonstrated significant improvements in third-order false belief understanding and rational/balanced mentalizing styles.

This study sheds light on the effectiveness of school-based interventions that target the enhancement of teachers’ abilities in emotional awareness, metacognition, and reasoning about mental states. It highlights how such improvements in teachers can have a positive and direct impact on students’ ToM, mentalizing, and metacognitive skills. Crucially, this study brings to the fore the potential scalability of teacher-centred training programs. Unlike intensive child-centric curriculums, teacher-focused interventions could offer a more feasible and resource-efficient means for broad implementation. This approach suggests a promising pathway for widespread adoption in educational settings, where the training of key adults - the teachers - can indirectly but effectively foster the socio-emotional and cognitive development of a larger number of students.

#### School-based interventions using a systems approach

A systems approach, which encompasses comprehensive interventions targeting relationships, climate, disciplinary policies, and individual competencies within schools, has been the focus of four significant studies. These studies have examined the impact of multi-component, school-wide interventions that apply a mentalizing lens to the entire school environment.

Through a series of studies, the Creating A Peaceful Schools Learning Experiment (CAPSLE) was implemented. Developed in 1992, this programme resulted in a Randomized Controlled Trial (RCT) encompassing nine elementary schools and over 3,600 pupils [11]. The CAPSLE programme integrates various interventions at different levels in the school system with the aim to foster a mentalizing culture. This approach involves recognizing and addressing instances of mobbing, where bullies, victims, and bystanders play different roles. The process engages pupils, teachers, and parents in reducing shame and punishment while promoting dialogue and perspective-taking. The results showed notable reductions in peer victimization, aggression, and disruptive classroom behaviour compared to control schools after two years.

In 2001, The effectiveness of the CAPSLE intervention was evaluated in a pilot study and implemented a comparable anti-violence intervention in an elementary school [55]. This intervention encompassed zero-tolerance policies, disciplinary procedures, and mentoring programs over one academic year, leading to reductions in suspensions, disciplinary referrals, and improvements in standardized test scores.

A few years later, an RCT comparing the effects of the School Psychiatric Consultation (SPC), CAPSLE, and treatment-as-usual among 1,345 third and fifth graders was conducted [19]. The teacher-implemented CAPSLE intervention was found to reduce aggression and improve classroom behaviour, specifically leading to a decrease in the number of children nominated by peers as aggressive or victimized.

Furthermore, an attachment and mentalizing theory-focused training program was delivered to all staff in a violent Jamaican high school over three years [20]. This intervention led to decreases in victimization and improvements in academic performance, school climate, and teacher retention compared to other regional schools. This methodologically sound study provides robust evidence for the effectiveness of the CAPSLE program in reducing disruptive and aggressive behaviours when applied at a whole-school level.

Synthesizing findings across these studies, it is evident that multi-faceted universal interventions that encompass mentalizing and relationship-focused training programs at the whole-school level are promising. Targeting school policies and disciplinary procedures alongside teacher practices and student competencies seems effective in bringing about systemic improvements. These include decreased suspensions and peer aggression, as well as enhanced school climate, teacher retention, and academic performance.

Collectively, these studies provide strong evidence supporting the benefits of school-based interventions grounded in mentalizing theory across developmental stages from middle childhood to adolescence. Interventions ranging from training in emotion understanding, empathy, social cognition, relationships, and school climate have demonstrated consistent improvements in socio-emotional competencies, classroom behaviours, and academic functioning. The generalizability of these interventions, from deaf students to violent schools across various countries, indicates their robustness.

However, only a few studies have assessed long-term outcomes, leaving questions about the sustainability of these effects. Despite this, the consistent pattern of positive effects from randomized trials presents compelling grounds for further development of mentalizing training to enhance social, emotional, and academic learning.

In conclusion, school-based interventions that focus on, promote, or are grounded in mentalizing theory have shown broad effectiveness in advancing core socio-emotional competencies such as emotion understanding, empathy, perspective-taking, and ToM among students. Associated benefits include enhanced classroom behaviours, teacher functioning, school climate, reduced peer aggression, and indications of improved academic and creative performances. These consistent results using programs that embed mentalizing components in curriculums, train teachers, or restructure whole-school systems underscore the value of continued research and broader implementation initiatives. Such efforts are vital for fulfilling schools’ potential in nurturing mentalizing capacities, which are fundamental for healthy development, relationships, and peaceful coexistence within schools and the wider communities they serve.

## Discussion

### Effectiveness for enhancing mentalizing and socioemotional competencies

The primary aim of this systematic review was to rigorously examine mentalizing-based school interventions targeted at children and adolescents, with a focus on those aged 6–18. Our goal was to synthesize thematically the findings from the studies we identified, providing insights into the effectiveness of these interventions.

The collection of studies reviewed offers encouraging initial evidence supporting the notion that school-based interventions, which integrate mentalizing components, are effective in fostering a range of capacities pertinent to mentalising. These capacities include emotional awareness, understanding, and regulation, as well as social cognition and perspective-taking, across childhood and into adolescence [37, 38, 40, 41, 43, 47, 49, 50]. The benefits observed in the interventions encompassed enhanced empathy, Theory of Mind (ToM), metacognition, improved classroom behaviour, well-being, functionality, and healthier peer relationships.

### Mental health and well-being

From a child development perspective, this review highlights mentalization-based interventions in schools as crucial for children’s development, guiding them through complex stages of growth encompassing emotional well-being, cognitive skills, social interactions, and their educational journey. The interventions aim to enhance children’s self-understanding and foster emotional regulation and resilience. The Resilience Program for example [33], strengthens resilience and mentalization in children and adolescents, using accessible metaphors to educate them about stress responses and mentalization. The program demonstrated potential, with staff continuing to employ its tools three years later. Mentalization-based methods offer cost-effective strategies to boost resilience, self-management, and relational skills. Other initiatives, such as the Peaceful Schools Program [19] and Emotion-Based Social Skills Training [42], combine mentalization, empathy, and emotional insight. Conversation-focused training is particularly effective in enhancing mentalizing abilities and social skills, with randomised controlled trials consistently reporting improvements in theory of mind (ToM) and social competencies.

### Social interactions and peer relationships

Theory of Mind (ToM) training programmes illustrate the impact of focused discussions on mental states, facilitated by teachers, on enhancing children’s social cognition and understanding of others. Improving ToM abilities in middle childhood is crucial for forming peer relationships, navigating social contexts, and attaining academic achievements [56]. Brief, specific conversations and training aimed at comprehending others’ mental states and perspectives can beneficially influence children’s socio-emotional development and competencies. Integrating such programmes into teaching practices presents a promising method for promoting socio-emotional development, supporting behavioural adjustment and well-being. Interventions centred on mental state discussions can enhance peer relationships and alleviate loneliness among children. Incorporating ToM-focused discussions into school curricula could foster socio-emotional development as children shift from family-centric to peer-centric social worlds [40]. The iterative nature of social perspective training [39], highlights its potential for broad impact on the school environment, as teachers and staff feel understood and offer empathetic understanding to students.

### Function

Interventions such as the creativity and divergent thinking training programme [38] and the study focusing on empathy through interaction with a virtual tutor agent [46] illustrate the impact on functioning. These interventions create strong support systems, recognising that optimal functioning is a collaborative process between individual capabilities and the quality of relationships and environments. The review points to the benefits from some universal interventions as well as programmes targeted at higher-risk groups [41, 55]. These interventions can improve mental state understanding and reasoning abilities, vital for positive socioemotional development and skills, aligning with research emphasising the role of mentalising in self-regulation, social functioning, and promoting harmonious relationships [57]. Well-designed training programmes, implemented at key developmental stages, can significantly enhance mentalising abilities, supporting healthy development for a wide segment of the student population.

### From theory to practice: MBIs in school settings

The findings of this systematic review identified a varied spectrum of interventions designed to meet the mentalizing needs of children. While a small proportion of these interventions cater to children with specific diagnoses, such as Autism Spectrum Disorder (ASD), the bulk of them target broader populations, often united by common contextual factors like vulnerability to social challenges [19, 55].

It is important to note that only a limited number of these interventions are explicitly categorized as mentalizing-based. Given the existing range of approaches, it is likely that a methodically developed MBIs, specifically designed for school settings, could yield significant benefits. Such an intervention would integrate various elements within the broad spectrum of mentalizing, including, but not limited to, Theory of Mind (ToM) and empathy.

The studies suggest that an effective MBIs in schools would ideally incorporate activities and exercises aimed at promoting ToM [37, 40, 44, 45, 47]. This aspect is critical for enhancing students’ capacity to understand social situations, interpret others’ perspectives, and cultivate positive interpersonal relationships. Additionally, incorporating components focused on empathy could substantially further contribute to the socio-emotional growth of students [51]. A well-structured program would offer students practical opportunities to develop and reinforce their empathic abilities, thereby nurturing a more compassionate and supportive school environment.

Furthermore, the role of teacher training in such interventions may be pivotal. A number of studies reviewed suggest that providing teachers with comprehensive training would enable them to effectively integrate mentalizing-based strategies into the school curriculum and day-to-day interactions with students [33, 39].

In summary, the diverse interventions identified in this review underscore the potential for a more focused and explicit MBT approach in schools. Such a program, specifically addressing key areas like ToM and empathy, could significantly enhance students’ social-emotional development, foster healthy relationships, and contribute to a positive learning atmosphere. Continued research and development in this area promise to make substantial contributions to the domain of school-based interventions and mental health promotion, underscoring the value of mentalizing as a core component in educational settings.

This contrasts with the trends observed in systematic reviews of MBT with adults, where the emphasis has predominantly been on specific diagnostic groups, including individuals with Borderline Personality Disorder, Eating Disorders, or Depression [58]. In contrast, the school environment offers a unique platform for interventions targeting young individuals without a formal diagnosis. This setting is conducive to implementing preventative strategies and addressing behavioural concerns early on.

### Generalization and sustainability

A crucial aspect to consider regarding the effectiveness of school-based mentalizing programs is the extent to which the socioemotional benefits observed are generalizable across different evaluators and sustainable over time. The evidence in this respect is somewhat varied. For example, in conversation-based Theory of Mind (ToM) interventions, two randomized trials [37, 44] reported improvements in strange stories task performance that were maintained over two-month follow-ups. However, a five-session program [40] noted that these gains diminished after two months. The social perception training model studied [50] did not assess the sustainability of its effects. In the realm of emotional awareness training, there was only one study that measured outcomes beyond the immediate post-test, finding sustained improvements in emotion comprehension at six months [47]. Studies examining teacher practices or whole-school programs, which typically spanned at least two years, did report sustained effects on aspects such as bullying, school climate, and academic scores [11, 20]. This suggests that for long-term effects to be ascertainable, such interventions may need to be integrated into the schools’ regular approach to education and its curricula.

In terms of generalisability, compelling evidence was presented in the Peaceful Schools programme, which achieved simultaneous reductions in peer-reported aggression, teacher-reported disciplinary problems, and observed classroom disturbances [11]. However, a disparity between teacher and parent assessments suggests that enhancements in socio-emotional skills observed within school settings might not completely extend to other environments without parallel family parenting training [42]. In many studies only a small number of classrooms were involved in the research limiting sample size and guarantee generalisability [37]. In a similar vein, promising findings from a sample sourced from only two communities, limit the external validity of the results [38]. These limitations highlight the critical need for replicating studies across more varied and representative samples to solidify the reliability and wider applicability of the outcomes of interventions.

Therefore, while the initial body of literature suggests that some MBIs can produce mentalizing gains associated with increased functioning and wellbeing that may be both sustainable and generalizable, there is a critical need for further research. This research should assess long-term outcomes and utilize multi-informant approaches to better understand the limitations and scope of these interventions. Identifying the active components and implementation factors that predict broader impacts, as opposed to those yielding isolated benefits, will be particularly valuable. This will help to isolate the most effective elements of mentalizing-based school interventions, guiding future efforts to maximize their efficacy and reach.

### Limitations and future research directions

The current research on mentalizing-based school interventions, while encouraging, has several limitations that should be acknowledged. One significant issue is the varying definitions, interpretations, and implementations of mentalizing in Mentalizing-Based Interventions (MBIs). This inconsistency complicates the comparison and synthesis of findings across studies. Future research should aim to standardize intervention protocols and outcome measures to enhance the comparability and generalizability of findings. Additionally, although interventions often successfully change social cognition, they do not necessarily lead to improvements in other domains, as might be expected. The failure to establish the mediation of benefits through improved mentalizing leaves questions about the mechanisms by which outcomes are achieved.

Some have questioned the relevance of mentalizing to all cultural groups [59, 60]. The lack of diversity in age groups and cultural contexts within existing studies calls for a more nuanced approach in tailoring interventions [59, 60]. Investigating the differential effectiveness of mentalizing interventions across developmental stages and cultural settings is essential for developing interventions responsive to diverse populations’ needs.

Limitations also arise from reliance on online searches, which may not capture all relevant literature, and the possibility of publication bias. The research question introduces challenges in defining precise search terms because the lack of maturity in this body of research and the lack of general agreement about what a mentalization focused intervention may be, distinguishing between explicitly MBIs and those aimed at improving selective aspects of social cognition. There are almost no replicated studies and many of the interventions are not sufficiently well described to permit replications. None of the studies were preregistered and prespecified data analytic approaches to testing hypotheses.

Future research should explore the implementation and impact of well-constructed, MBIs designed for school settings, encompassing Theory of Mind, empathy, and perspective-taking. Investigating the effectiveness of providing teachers with structured training to integrate these interventions into the school environment and assessing the fidelity with which such interventions are delivered may be a promising line for future assessment of MBIs.

Assessing downstream functional impacts of MBIs in schools, particularly in relation to academic achievement, absenteeism, and graduation rates, could establish the relevance of the approach. Observational tools evaluating the application of trained mentalizing skills in real-world settings could provide further valuable insights.

We have not identified qualitative and mixed-methods approaches which should be employed to capture participants’ experiences, shedding light on key mechanisms, barriers, and contextual factors supporting implementation and efficacy.

Also, cost-effectiveness analyses weighing expenses against monetized benefits in areas such as lifetime productivity, healthcare utilization, or juvenile justice involvement could provide incentives for large-scale investment and policy support, particularly if findings indicate favourable returns on investment.

### Summary and conclusion

Despite certain inevitable constraints, the existing body of research offers some initial evidence that mentalizing training initiatives, when well designed and timed developmentally, can be effective tools for enhancing the socioemotional competencies crucial for healthy development, learning, and peaceful interactions among schoolchildren. The collective findings from studies on mentalizing-based school interventions underscore their capacity to positively influence both cognitive and socio-emotional development. Although there are methodological variations and challenges, the overarching trend indicates that the incorporation of mentalizing practices into educational settings is a promising approach for nurturing individuals who are resilient, empathetic, and academically successful.

Programs centred around conversation-based activities have shown particular potential for cost-effective enhancement of emotional awareness, understanding, and social cognition, especially when integrated into the regular curriculum and the broader school culture. With a future emphasis on increased methodological rigour to clarify boundary conditions and assess the additive effects alongside other evidence-based programs, the current evidence supports the continued refinement, optimization, and scaled implementation of initiatives that promote mentalizing. These programs have the potential to address both the socioemotional needs and core academic functions of contemporary educational systems.

The feasibility of implementing mentalizing training programs across various countries, developmental periods, and educational settings further enhances their appeal. It underscores the potential of these programs to support central educational goals globally, such as fostering empathy, perspective-taking, and emotional intelligence. This aligns with the broader aim of educating a global citizenry equipped with the essential skills for emotional understanding and effective social interaction. The implications of this research extend beyond the confines of individual classrooms or schools, suggesting a significant impact on the development of empathetic and socially adept individuals who can contribute positively to their communities and society at large.

## Data Availability

All data for this review is held by the first author (GCD).
